# Autocrine, paracrine and necrotic NMDA receptor signalling in mouse pancreatic neuroendocrine tumour cells

**DOI:** 10.1098/rsob.170221

**Published:** 2017-12-20

**Authors:** Hugh P. C. Robinson, Leanne Li

**Affiliations:** 1 Department of Physiology, Development and Neuroscience, University of Cambridge, Downing Street, Cambridge CB2 3EG, UK; 2 David H. Koch Institute for Integrative Cancer Research, Massachusetts Institute of Technology, 500 Main Street, Cambridge, MA 02142, USA

**Keywords:** pancreas cancer, single-channel recording, glutamate diffusion

## Abstract

*N*-Methyl-d-aspartate receptor (NMDAR) activation is implicated in the malignant progression of many cancer types, as previously shown by the growth-inhibitory effects of NMDAR antagonists. NMDAR-mediated calcium influx and its downstream signalling depend critically, however, on the dynamics of membrane potential and ambient glutamate concentration, which are poorly characterized in cancer cells. Here, we have used low-noise whole-cell patch-clamp recording to investigate the electrophysiology of glutamate signalling in pancreatic neuroendocrine tumour (PanNET) cells derived from a genetically-engineered mouse model (GEMM) of PanNET, in which NMDAR signalling is known to promote cancer progression. Activating NMDARs caused excitation and intracellular calcium elevation, and intracellular perfusion with physiological levels of glutamate led to VGLUT-dependent autocrine NMDAR activation. Necrotic cells, which are often present in rapidly-growing tumours, were shown to release endogenous cytoplasmic glutamate, and necrosis induced by mechanical rupture of the plasma membrane produced intense NMDAR activation in nearby cells. Computational modelling, based on these results, predicts that NMDARs in cancer cells can be strongly activated in the tumour microenvironment by both autocrine glutamate release and necrosis.

## 1. Introduction

The *N*-methyl-d-aspartate receptor (NMDAR) is an ionotropic glutamate receptor which is present at most excitatory glutamatergic synapses in the nervous system [1,2]. It is calcium-permeable, but blocked in a voltage-dependent way by extracellular magnesium ions. In the central nervous system (CNS), these properties enable an activity-dependent switch, turning on downstream kinases and changes in gene expression which regulate synaptic efficacy [3], neuronal proliferation and survival [4,5]. Functional NMDAR signalling has been reported in tissues outside the CNS, for example in skin keratinocytes, during re-epithelialization following skin injury [6]. Expression of NMDARs has also been found in a variety of human cancer cell lines [7], and in human small-cell lung cancer and breast cancer tumours, whose growth in mouse xenografts is inhibited by NMDAR blockers [8,9], as well as in prostate cancer [10].

Although NMDAR expression has been detected by qRT-PCR or western blotting, the electrophysiology of these ectopically-expressed NMDARs in cancer cells has not been elucidated. Do NMDARs mediate significant membrane current and calcium influx? What are the sources of their main ligand, glutamate, for their activation in cancer cells? Are NMDARs activated by autocrine or paracrine glutamate secretion, or both? Using the RIP1-Tag2 mouse model of pancreatic neuroendocrine tumour (PanNET) [11], Li & Hanahan [12] showed that NMDAR activation is associated with invasive tumour growth, and proposed a model in which autocrine release of glutamate, stimulated by interstitial fluid flow, causes self-sustaining, autologous NMDAR activation in cancer cells. Here, we take advantage of a cancer cell line derived from this GEMM, and use low-noise whole-cell patch-clamp recording to illuminate the electrophysiology of NMDARs in cancer cells.

## 2. Results

### 2.1. Whole-cell patch-clamp reveals functional NMDAR ion channels in a mouse model of PanNET

**Figure 1.**
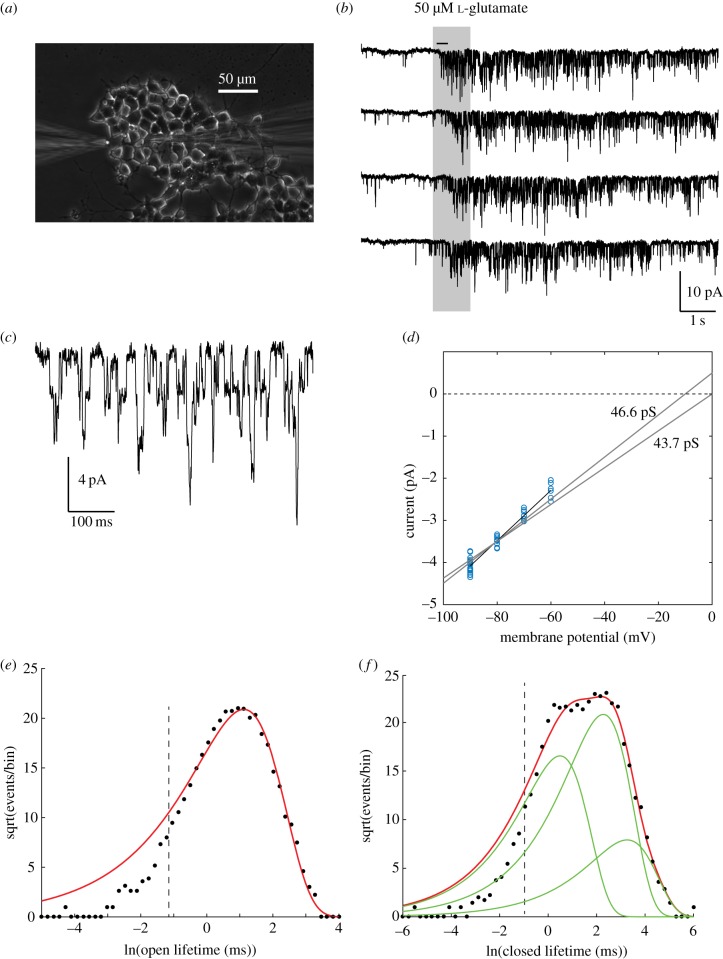
Functional NMDARs in PanNET cells. (*a*) Puff application of glutamate. Puffer pipette at left, patch pipette at right. (*b*) Whole-cell patch recording, 500 Hz Gaussian digital low-pass filter, −80 mV. (*c*) Expanded segment of top trace in *(b)* (indicated by horizontal bar) showing single-channel opening and closing transitions. (*d*) Current–voltage plot of channel openings. Each point is data from one cell at one holding potential (*n* = 21 cells). Best-fit slope conductance (*γ*) for reversal potential (*E*_rev_) of 0 mV was 46.6 pS; and for *E*_rev_ = −5 mV, *γ* = 43.7 pS. (*e*,*f*) Sigworth-Sine plots [13] of channel lifetime distributions in one cell. (*e*) Open-state durations fitted to a single exponential distribution (6129 events, bin size = 0.18, missed events cut-off < 0.331 ms indicated by dashed vertical line). *τ* = 3.06 ms. (*f*) Closed-state durations, fitted to a sum of three exponential components (8183 events, bin size = 0.24, *a*_1_ = 0.318, *τ*_1_ = 1.63 ms, *a*_2_ = 0.595, *τ*_2_ = 9.75 ms, *a*_3_ = 0.09, *τ*_3_ = 26.0 ms.

We carried out whole-cell patch-clamp recordings (figure 1*a*) in a tumour cell line (βTC-B6) derived from the RIP1-Tag2 transgenic mouse model of PanNET bred into the C57BI/6 (B6) strain [12]. Using internal (high K^+^) and external solutions mimicking physiological ionic conditions, we found that these cells were excitable in current-clamp, had high input resistances (2–8 GΩ), membrane capacitances of 5–30 pF, and major inward and outward voltage-dependent currents whose kinetics were consistent with the predominant L- and N-type voltage-gated calcium and Kv2.1 delayed rectifier potassium currents of normal pancreatic β cells [14]. Next, we used an intracellular solution in which potassium was replaced by caesium, to reduce baseline noise [15] and allow single-channel resolution in the whole-cell current [16], and magnesium was omitted from the extracellular solution, to prevent magnesium block of NMDARs. Application of l-glutamate or NMDA caused agonist-activated channel openings (figure 1*b*,*c*), whose amplitude (40–50 pS), reversal potential (≈0 mV, figure 1*d*) and open and closed lifetime distributions figure 1*e*,*f*) coincide with those expected of GluN2A/B-containing NMDA receptors [17], with a mean open time of 3–5 ms, and a complex closed-time distribution with at least three exponential components.

### 2.2. Intracellular calcium activity is influenced by NMDARs

**Figure 2.**
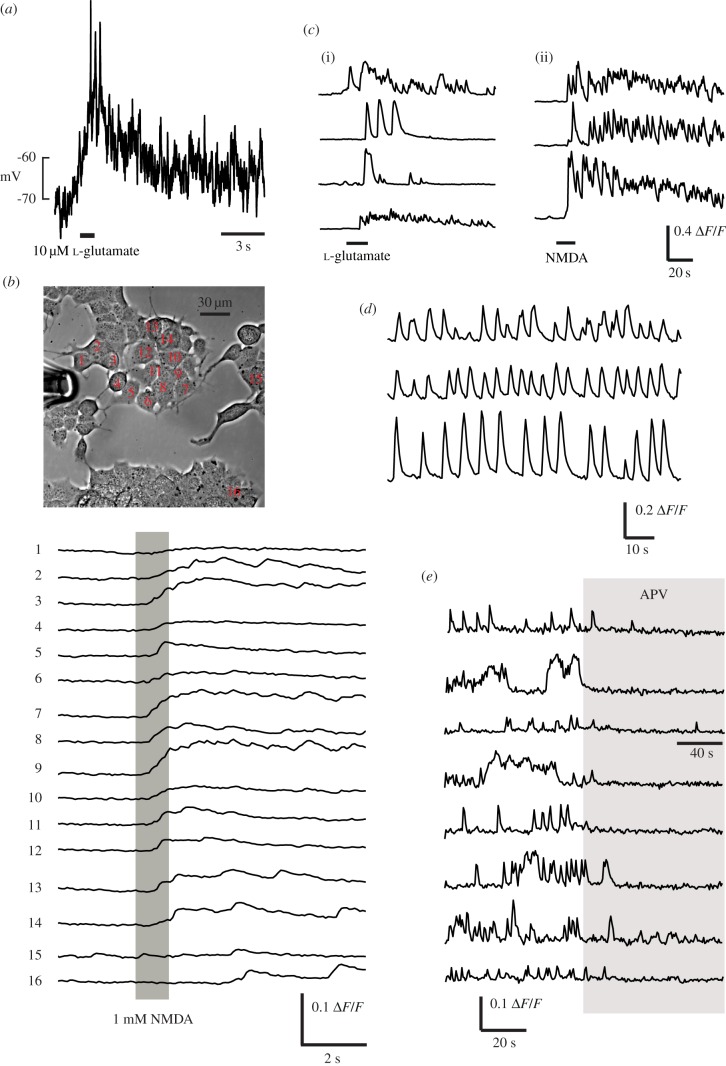
NMDARs can stimulate membrane depolarization and calcium influx. (*a*) whole-cell current-clamp recording with high [K^+^] solution in the pipette. A 1s pulse of l-glutamate containing Ringer solution (500 µM) was applied through a perfusion pipette at the time indicated, causing a large, maintained depolarization accompanied by action potentials. (*b*) After loading with Oregon Green BAPTA-1AM, fluorescence was recorded while puff-applying 1 mM NMDA dissolved in the Ringer solution. Synchronous elevations in intracellular calcium are observed in cells across the field which are exposed to the specific agonist. 90 of 173 cells analysed (52%) showed detectable responses (average Δ*F*/*F* 6.44% in responding cells) to pressure ejection of glutamate or NMDA. (*c*) Example calcium responses in several cells to (i) 20 µM l-glutamate or (ii) 1 mM NMDA. (*d*) Three simultaneously-recorded cells showing spontaneous calcium transients, at 35°C in 1 mM Mg^2+^ extracellular solution. (*e*) Examples of APV-induced silencing of spontaneous activity in cells from eight different culture dishes. Perfusion with 1 mM APV occurs during the period indicated by the grey bar.

As described in a separate study [18], activating NMDARs in these PanNET cells caused depolarization and calcium influx (figure 2*a*,*b*), confirming that βTC-B6 cells express functional NMDARs which are capable of depolarizing cells to firing threshold and causing calcium influx. Here, we extended these observations by showing that sustained calcium activity can also be initiated by l-glutamate or NMDA (figure 2*c*(i,ii) in a physiological concentration (1 mM [19]) of extracellular Mg^2+^. We also often observed repetitive spontaneous calcium transients, in zero or 1 mM Mg^2+^, both at room temperature or near-physiological 35°C (figure 2*d*). We investigated the effect of applying APV (50 or 100 µM) to regions of cultures showing ongoing spontaneous calcium activity. Although APV did not always block spontaneous calcium activity, we found multiple regions where activity was clearly silenced with the onset of perfusion (figure 2*e*). This indicates that NMDARs contribute to and, in some cases, drive spontaneous activity, which may also be sustained by other sources of intrinsic excitation.

### 2.3. NMDARs are activated by paracrine and autocrine release of glutamate

Glutamate is estimated to be present in the cytoplasm of neuronal cells at a concentration [Glut]_i_ of 1–10 mM, with approximately 100 mM concentrated in synaptic vesicles [20]. In pancreatic β cells, intracellular glutamate concentration has been estimated to be 1–7 mM [21]. The most direct measurements of cytosolic glutamate in living cells have been carried out using ^13^C NMR spectroscopy in proliferating glioblastoma cells, showing it to have a level of around 20 mM [22].

In morphologically-compact cells such as βTC-B6, the intracellular compartment is well-perfused in the whole-cell recording mode, and freely-diffusing glutamate should equalize in concentration between the ≈15 µl of pipette solution and the ≈2 pl of cytoplasm within seconds [15]. Therefore, to assess autocrine activation, a physiological level of free cytoplasmic glutamate should be included in the pipette filling solution, to allow normal rates of glutamate release [23]. In contrast, a glutamate-free pipette solution effectively clamps cytoplasmic glutamate concentration to zero: thus, spontaneous NMDAR channel openings observed in this condition will reflect only paracrine activation, since direct efflux or vesicular packaging of glutamate would not be maintained.

**Figure 3.**
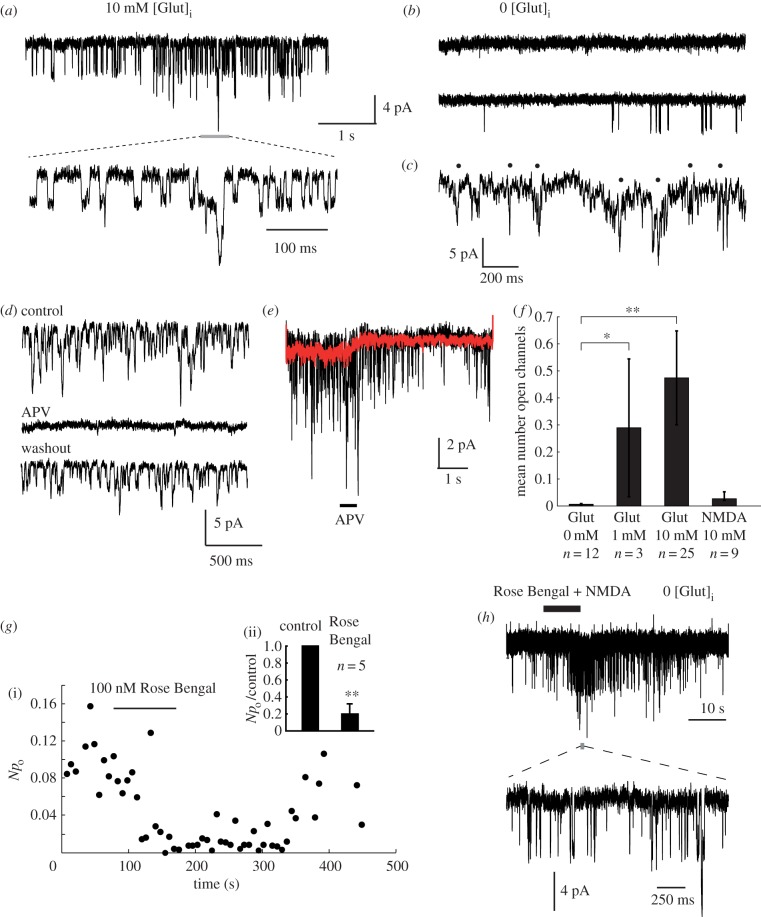
Autocrine activation of NMDARs. (*a*) A constant NMDAR channel activation is observed at −90 mV using pipettes filled with a caesium gluconate based solution containing 10 mM l-glutamate (autocrine condition). A segment of channel activity at higher time resolution is shown below, as indicated. (*b*) Very infrequent or no NMDAR activation is seen when glutamate is omitted from the patch pipette (paracrine condition). (*c*) Nystatin-perforated patch recordings show poorly-resolved 4–5 pA openings (indicated by black spots). (*d*) Channel activity is completely and reversibly blocked by perfusion with 1 mM APV. (*e*) Puff-perfusion of APV solution for 500 ms causes a rapid block in channel openings. Black trace: individual trial. Red trace: ensemble average of 20 successive trials. (*f*) Summary of the channel activity produced by intracellular agonists. The ordinate is the average number of open channels measured over a 5 s period at −90 mV. Significance: control versus 1 mM Glut, *p < 0.0484; control versus 10 mM Glut, **p < 0.000026; control versus 10 mM NMDA, *p* > 0.458; Wilcoxon rank sum test. (*g*) Sensitivity of autocrine NMDAR activation to a VGLUT inhibitor: (i) Reversible block of autocrine activity by 100 nM Rose Bengal. *Np*_o_ is the product of channel number and open probability, equivalent to the mean number of simultaneously-open channels. (ii) Rose Bengal (100 nM) block is significant, **p < 0.0079, Wilcoxon rank test, *n* = 5 cells. (*h*) Rose Bengal (100 nM) does not block responses to exogenously applied NMDA (1 mM) in cells recorded with zero glutamate in the pipette (paracrine condition). A short segment of channel activity is shown at high time resolution below, as indicated.

Addition of 1 or 10 mM l-glutamate to the caesium-based pipette solution (figure 3*a*) caused a striking activation of NMDARs (figure 3*a*) in most cells, which showed the characteristic ≈45 pS slope conductance, and could be blocked by perfusion with APV, a specific antagonist of NMDARs (figure 3*d*,*e*). All detectable openings were also abolished by 1 mM [Mg^2+^]_o_ (*n* = 10 cells, not shown). In contrast, when glutamate was omitted from the pipette solution, only sparse or no channel openings were seen (figure 3*b*). We also carried out nystatin perforated-patch recordings (see Material and methods) in order to test for NMDAR opening by endogenous cytosolic glutamate. With this technique, the signal-to-noise level was far lower, but 4–5 pA inward channel openings were apparent at a holding potential of −90 mV (figure 3*c*, five cells). We quantified channel activity by measuring the average number of simultaneously-open NMDAR channels over periods of 30–60 s (figure 3*f*). 1 mM [Glut]_i_ produced about 60% of the activation level for 10 mM [Glut]_i_. This activation was specific for glutamate, as 10 mM [NMDA]_i_ did not significantly elevate the channel open probability over control. We conclude that this elevated NMDAR activity represents autocrine signalling, with release occurring through a glutamate-specific transport pathway, and that occasional openings observed with zero-glutamate intracellular solution represent paracrine activation by glutamate released from other, nearby cells.

To identify the route by which glutamate exits the cell to cause autocrine activation, we investigated the effect of specific blockers of glutamate transport on autocrine activity. Major candidates for pathways of autocrine glutamate release include the excitatory amino acid transporters (EAATs) [24], the system x_c_^−^ cystine/glutamate antiporter [25], and the vesicular glutamate (VGLUT) transporters [26], which are known to be expressed in this cell line [12]. Sulfasalazine, a potent (*K_i_* < 1 µM) and specific blocker of system 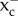, and dl-threo-β-benzyloxyaspartate (DL-TBOA), a specific blocker of EAATs, failed to reduce channel activity at concentrations of 10 µM (*n* = 4 cells) and 100 µM (*n* = 6 cells) respectively. In contrast, Rose Bengal [27], which is a highly potent, noncompetitive, membrane-permeable blocker of VGLUTs (*K_i_* = 19 nM), effectively blocked NMDAR currents at 100 nM (*n* = 5, figure 3*g*), and the blockade was reversible within several minutes following application. In contrast, Rose Bengal did not block responses to exogenously-applied NMDA (figure 3*h*). We also tested another VGLUT blocker that is less membrane-permeable, Trypan Blue [28], and found that it reduced opening probability irreversibly to 28% and 18% of control in two cells, but had no effect in two other cells, possibly due to failure of the compound to access an intracellular site of action. These data suggest that the release of glutamate across the plasma membrane is dependent on VGLUTs, but nonspecific effects of these inhibitors on some other process of glutamate release cannot be ruled out.

**Figure 4.**
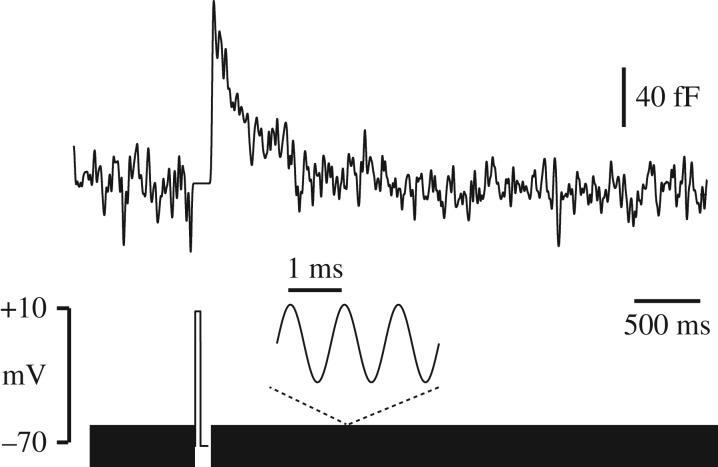
Depolarization triggers capacitance transients indicative of vesicular release. Membrane capacitance was monitored by applying a 1 kHz sinusoidal voltage command (bottom). A brief (100 ms) depolarizing step produces a fast rise in membrane capacitance, followed by a slow decline back to its baseline level. Similar results were seen in eight cells.

By monitoring cell membrane capacitance using a 1 kHz sinusoidal voltage-clamp [29], we were able to detect the rapid fusion and slower subsequent resorption of vesicles with the plasma membrane, in response to depolarizing steps in voltage-clamp, similar in size and duration to action potentials (figure 4). There is scope therefore for a vesicular mechanism for glutamate release, as proposed by Li & Hanahan [12], which might be at least partially under the control of membrane potential. However, exocytosis dependent on voltage-gated calcium channel opening is unlikely to be triggered at potentials below −70 mV, and voltage-dependence of autocrine-activated NMDAR current was not significant over the measured range −90 mV to −70 mV (not shown). We cannot rule out that channel activity might be voltage-dependent above −60 mV, as this is outside the potential range in which we can adequately resolve single channels in the whole-cell current.

### 2.4. Properties consistent with GluN2B-containing NMDARs

**Figure 5.**
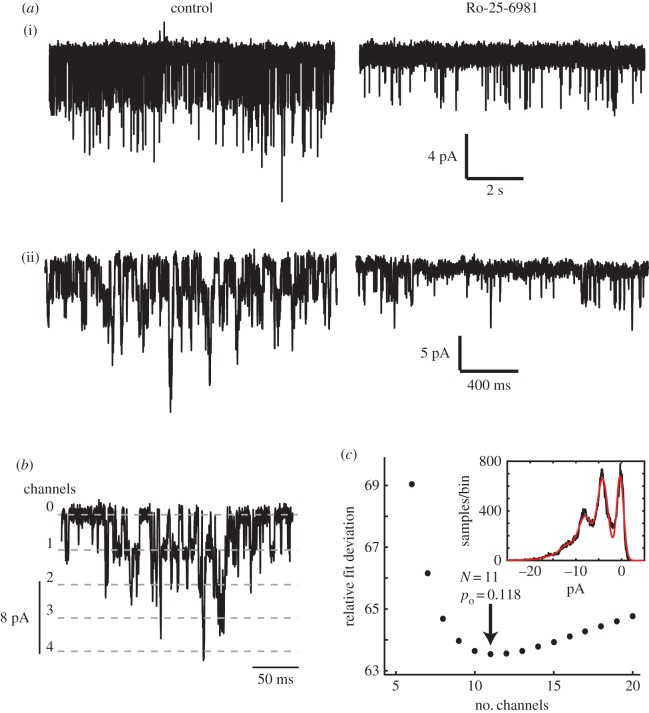
Properties characteristic of GluN2B-containing NMDARs. (*a*) Autocrine-activated NMDAR openings are inhibited by a GluN2B-specific blocker. (i), (ii): examples in two different cells of the effect of applying 50 µM Ro-25-6981 (right) versus control (left). On average, open probability was reduced to 14% of control in four cells. (*b*,*c*) High probability opening of NMDARs during autocrine activation. (*b*) 500 ms epoch of autocrine-activated channel openings, at a holding potential of −90 mV, illustrating multiple channel open levels. (*c*) Amplitude histograms (inset) were fitted by a binomial model for a population of *N* independent and identical channels each open with a constant probability *p*_o_. In this example, optimal fit is obtained for *N* = 11, *p*_o_ = 0.118.

The functional NMDARs in this cell type appear to be predominantly GluN2B-containing, based on the following evidence. Firstly, their high conductance (40–50 pS) is typical for GluN2A or GluN2B, versus GluN2C/D channels. Second, openings were largely blocked by applying the GluN2B-selective antagonist Ro 25-6981 (figure 5*a*, *n* = 4 cells). These findings are consistent with the high GluN2B expression reported in this cell line [12]. It is possible that the residual openings seen in the presence of Ro 25-6981 correspond to GluN2A-containing receptors, as GluN2A is also expressed in these cells, and would have an identical single-channel current amplitude.

In addition, we estimated the open channel probability of NMDARs during autocrine activation by measuring the relative likelihood of multiple simultaneous channel openings (figure 5*b*,*c*). Assuming a functional population of *N* identical and independent NMDARs, each opening with probability *p*_o_, the frequencies of multiple simultaneous openings should follow a binomial distribution [30]. We fitted current amplitude histograms with a multi-Gaussian distribution, in which the integral of each peak was proportional to the corresponding binomial probability, for a range of different assumed *N* values, larger than or equal to the maximum number of simultaneously observed open channels (figure 5*c*). In six out of seven cells analysed, the least-squares fit showed an optimum at a small finite value of *N*, which can be taken as the size of the population of activated receptors. In these six cells, the mean *N* was 7, and the mean open probability *p*_o_ was 0.107. In the remaining cell, no optimum could be found: the fit improved progressively with increasing *N* (up to 40), effectively following a Poisson distribution—the limit of a binomial model for high *N* and low *p*_o_.

Notably, a value of *p*_o_ ≈ 0.1 coincides with the maximal value observed for GluN2B-containing channels when fully occupied by glutamate [31–34]. The EC_50_ for glutamate activation of GluN2B-containing channels is around 1.5 µM, and the Hill coefficient is about 1.5 [35], suggesting a lower bound for the glutamate concentration experienced by these channels in the region of 5–10 µM (e.g. the receptor should be 90% activated at 6.5 µM).

### 2.5. Rupture-induced necrosis releases large amounts of glutamate

Having established autocrine glutamate release as a potent mechanism of activating NMDARs in βTC-B6, we further asked if there could be another source of NMDAR-activating glutamate in the tumour microenvironment, both in PanNET and in other types of NMDAR-expressing cancer cells which may or may not co-express VGLUTs. We were particularly interested in the effect of necrotic cell death within the tumour microenvironment, as the poor blood supply in rapidly-growing tumours can lead to extensive necrosis. In contrast to apoptosis, in which cell contents are digested in an orderly way by surrounding cells, necrotic cell death is defined by abrupt rupture of the plasma membrane with unrestricted release of the cell contents. Necrosis can be either unregulated, where the plasma membrane is ruptured abruptly by physical forces, or regulated by specific signalling pathways, as in hypoxia-induced necrosis in tumours [36]. Since intracellular free glutamate is expected to be on the order of 1–10 mM, as discussed above, then the diluted contents of a single cell could in principle fill an extracellular volume 10^3^–10^4^ times larger with glutamate at a near saturating concentration for GluN2B-containing receptors. Glutamate released from necrotic cells could therefore be an extremely potent driver of invasion-promoting NMDAR signalling in a tumour.

**Figure 6.**
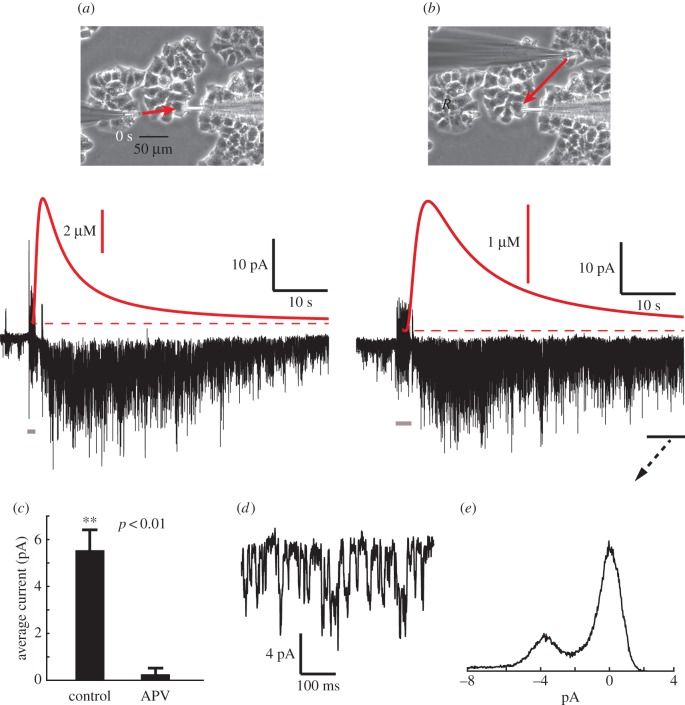
Cell membrane rupture activates NMDARs. (*a*) Whole-cell current (black waveform, below) recorded by pipette at right (photo at top), while a cell to the left undergoes necrosis, ruptured by driving an empty glass microprobe rapidly through the cell (see electronic supplementary material, video S1). The moment of rupture is indicated by the short grey bar at bottom. The distance between the two cells is about 80 µm, and the red arrow represents the flux of released cytoplasmic contents from the ruptured cell. A large activation of NMDARs ensues, subsiding over tens of seconds. For comparison, the putative time course of glutamate concentration at the recorded cell is shown above in red, from the solution of the 3D radial diffusion equation for the instantaneous point release of 2 × 10^−14^ moles of glutamate at a distance of 80 µm (see Material and methods). This quantity corresponds to a cell with a volume of 2 pl, containing 10 mM l-glutamate. Recording pipette is filled with high caesium, zero glutamate internal solution (paracrine condition). (*b*) Subsequent response in the same cell recorded while rupturing another cell, located in a different cell cluster, at a distance of 120 µm, with calculated glutamate transient in red. Membrane potential is −90 mV. (*c*) Comparison of the mean amplitude of a 1 s segment around the peak of the response, for control (*n* = 15 cells) and in the presence of 50 µM APV (*n* = 3 cells), showing a near complete block. (*d*) Detail of the decay phase of (*b*), within the period indicated by the black bar. (*e*) Current amplitude histogram of the indicated segment of (*b*), showing that the current is composed of characteristic ≈4 pA openings.

Using βTC-B6 as a model system, we tested this idea by recording paracrine NMDAR activation ([Glut]_i_ = 0) in one cell while rupturing the membrane of another cell in its neighbourhood. A glass micropipette, with a fire-polished tip, was positioned touching a target cell, abruptly advanced into and through the cell for a period of 1 s, then withdrawn, using a piezoelectric manipulator to step the position of the probe (see electronic supplementary material, video S1). This caused clear necrosis of the impaled cell, and simultaneously a large transient of inward current in the recorded cell (figure 6*a*). Necrosis responses could be recorded multiple times in the same cell by sequentially rupturing different neighbouring cells (figure 6*b*). For ruptured cells within a radius of 150 µm an average peak current of 5.54 ± 0.85 pA (s.e.m., 15 cells) was measured, with channel activation persisting for at least a minute. We calculated the timing and amplitude of the rupture-induced glutamate transient (see Material and methods), and this correlated well with the time course of channel activity (figure 6*a*,*b*, red traces). We confirmed that the necrosis response could be blocked by simultaneous perfusion of APV (figure 6*c*), and amplitude histogram analysis showed that the current was largely composed of ≈4 pA openings (figure 6*d*,*e*). In addition to glutamate, intracellular free aspartate, another effective NMDAR agonist, which has been estimated at around 1 mM in glioblastoma cells [22], may also contribute to this necrosis-induced NMDAR current.

**Figure 7.**
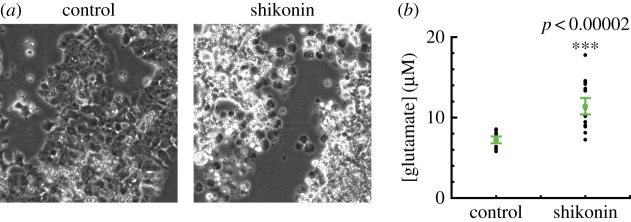
Shikonin-induced necroptosis causes a large release of glutamate from PanNET cells. (*a*) Appearance of cultures after 6 h incubation with control DMSO vehicle only (left) or 20 µM shikonin (right). Scale bars 100 µm. Extensive cell death and necrotic cell residue ‘ghosts’ are induced by shikonin. (*b*) Measurements of glutamate concentration in 1 ml of supernatant, after 90 min incubation of ≈300 000 cells in 20 µM shikonin (*n* = 16, ***p < 0.00002, Wilcoxon rank sum test).

To confirm the ability of necrosis to elevate extracellular glutamate concentration, we used the compound shikonin to induce programmed necrosis of βTC-B6 cells. Shikonin causes RIP1- and RIP3-dependent necroptosis by blocking pyruvate kinase M2 in tumour cells [37]. Incubation of cells in 20 µM shikonin caused extensive cell death (figure 7*a*); many necrotic ‘ghost’ cells were apparent within six hours. We then measured extracellular glutamate concentration relative to control using a fluorescence-based enzymatic assay (see Material and methods), and found that shikonin-induced necroptosis is associated with a large release of glutamate into the extracellular space (figure 7*b*). The average increment in glutamate concentration for 300 000 cells ml^−1^ was 4.19 µM (*n* = 16) relative to the DMSO vehicle control. This corresponds well with a predicted value of 6 µM, assuming a cell volume of 2 pl, a cytoplasmic concentration of 10 mM, and necrosis of the entire cell population.

## 3. Discussion

### 3.1. Functional NMDAR ion channels in cancer cells

NMDAR expression has been observed in various types of cancer, along with other glutamate receptors, but functional validation has largely been limited to demonstrating the effect of receptor blockade on cell survival. So far, electrophysiological evidence for functional NMDAR expression in cancer cells is only available in a handful of studies [7,9,38], with only one study examining detailed single-channel properties, of GluN2C-containing NMDARs in a phaeochromocytoma line (PC12) [38]. Here, by resolving single-channel currents in whole-cell recordings, we show the first direct electrophysiological evidence for autocrine and paracrine NMDAR signalling in cancer cells, as well as the first recordings, to our knowledge, of NMDAR channel activation by necrotic rupture of surrounding cells, in any cell type.

Clearly, NMDAR signalling in tumours *in vivo* will involve a much more complex 3D environment, interactions with other cell types in the tumour microenvironment, blood flow and interstitial fluid flow. Nevertheless, as a first step, it is important to understand quantitatively how it operates in a simplified 2D culture. In our experimental condition, βTC-B6 PanNET cells grow as an adherent monolayer, with much more area in contact with the underlying substrate than with neighbouring cells. If the glutamate release is uniformly distributed over the membrane, then it is clear that accumulation of glutamate will be higher underneath the cell, against the substrate, than on the upper surface. We used a computational model assuming steady, uniform glutamate release and simple diffusion to examine how glutamate might accumulate in this situation (see electronic supplementary material, figure S1 for details). This predicts that to raise the glutamate concentration in the restricted extracellular compartment beneath a cell to a level required to fully activate NMDARs (5 µM) would require a release rate of only around 10^−5^ fmoles/s per µm^2^ of cell membrane, equivalent to about 0.02% of the cellular content of free glutamate per second, assuming 10 mM cytosolic glutamate concentration, and a cell volume of 2 pl. In the whole-cell patch clamp, glutamate concentration is clamped by the relatively massive reservoir of solution in the patch pipette, but in the unperturbed cell, any net efflux would have to be compensated by resynthesis.

### 3.2. Mechanism of release of glutamate

Vesicular release of insulin is well-documented in normal pancreatic β cells [39], and glutamate has been reported to be packaged into insulin-containing vesicles by VGLUT3 [40,41]. If glutamate release in βTC-B6 cells were vesicular, and vesicles are assumed to be 100 nm in diameter (e.g. large dense-core vesicles are 300 nm diameter, synaptic vesicles typically 50 nm) and filled with 100 mM glutamate, then this level of release would require only 10 vesicles/cell/second, equivalent to 10 fF of membrane capacitance to be cycled every second (1 µm^2^ has a capacitance of ≈10 fF). This is just below the resolution of the technique for monitoring cell capacitance, although depolarization-triggered capacitance transients were readily resolved by ensemble averaging (figure 4). We were able to block the autocrine-activated NMDAR openings with Rose Bengal (figure 3*g*), suggesting that it depends on VGLUTs. It is unlikely, however, to be dependent upon conventional calcium-triggered vesicular release, as we found no voltage-dependence of channel activation over the range −100 to −60 mV (as noted earlier, background noise prevents resolution of NMDARs above about −60 mV). It is possible that there is spontaneous calcium-influx-independent vesicular release, as in cortical neurons [42]. A considerable fraction of VGLUTs in central neurons is found in the plasma membrane [43], and thus an alternative scenario is that plasma membrane VGLUTs might conduct intracellular glutamate down the large electrochemical gradient to the extracellular space [26]. In either case, the millimolar cytosolic concentration of glutamate could be maintained by its close coupling to the tricarboxylic acid cycle through alpha-keto-glutarate, and glutamine metabolism, which is an important source of energy in cancer cells [22]. In this connection, the insensitivity of intracellular glutamate level in PC12 cells to extracellular glutamate level suggests that intracellular glutamate is tightly regulated by metabolic processes, rather than being determined by plasma membrane glutamate transport [44].

### 3.3. NMDAR activation by necrotic release of glutamate

Our observation of very strong NMDAR responses to mechanical trauma-induced necrosis of nearby cells demonstrates an interesting mechanism of paracrine glutamate signalling, which may be of particular significance in cancer. Necrosis is frequently observed in some cancer types, and is often associated with a more aggressive disease and worse prognosis. It can occur as a result of the poor blood and energy supply in the tumour core, and can also be caused by some types of chemotherapy. We envision that glutamate release by necrotic cells in the tumour centre could be delivered by diffusion and interstitial fluid flow to the NMDAR-expressing cells at the tumour margin, thus further promoting tumour growth and invasion. It is quite likely that necrotic tumour interstitial fluid contains enough glutamate to produce a saturating activation of NMDARs. Recently, Eil *et al.* [45] found that tumour necrosis raises [K^+^] in tumour interstitial fluid (TIF) by about 7.5-fold over serum concentration, as cell rupture dumps the cytoplasmic contents of necrotic cells into the extracellular space, and the resulting raised [K^+^] inhibits T cell effector function within the tumour. A calculation, assuming that normal intracellular [K^+^] is 155 mM, and normal extracellular [K^+^] is 5 mM [30], predicts that about 1/6 of TIF in their study would originate from the cytoplasmic contents of necrotic cells. Therefore, the glutamate concentration of similar TIF might be on the order of 1 mM, in comparison with a *K*_d_ for glutamate binding to GluN1-GluN2B NMDARs of 1.5 µM. Interestingly, NMDAR expression is also seen in prostate cancer [10], and metastatic prostate cancer often becomes neuroendocrine-differentiated [46]. Raised serum glutamate concentration, which might reflect tumour necrosis, has been associated with prostate cancer aggressiveness and Gleason score, and suggested as a possible metabolic biomarker [47].

### 3.4. Computational model of NMDAR current in mPanNET cells

The key feature of NMDARs which allows them to serve as an ‘AND’ gate controlling synaptic plasticity is their magnesium block and consequent voltage-dependence, such that only the conjunction of presynaptic glutamate release and postsynaptic depolarization can trigger sufficient calcium influx for plasticity [3]. Although the majority of our experiments were carried out with nominally zero extracellular magnesium, in order to be able to optimally detect and study the NMDARs, we confirmed that both glutamate and NMDA can trigger repetitive calcium activity in physiological (1 mM) extracellular magnesium (figure 2*c*). Moreover, ongoing repetitive spontaneous calcium activity is also seen in 1 mM magnesium at near-physiological temperature (figure 2*d*).

**Figure 8.**
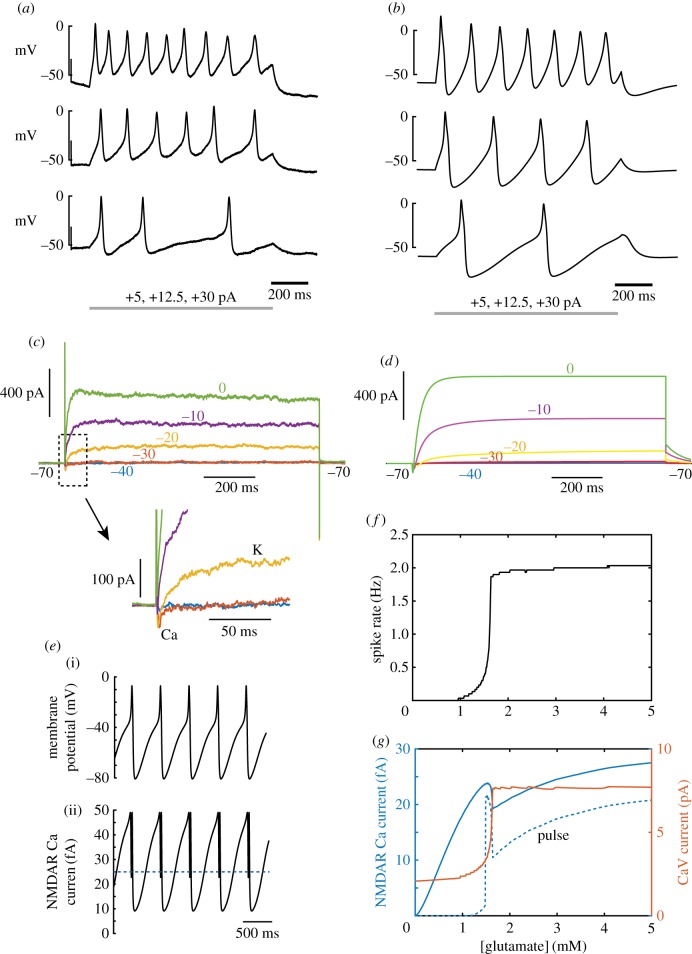
Effect of excitability and NMDAR voltage-dependence on calcium influx in a computational model. (*a*) Experimentally-recorded action potential firing in response to injection of increasing levels of current in current-clamp. (*b*) Corresponding responses for the model, containing 11 NMDARs. (*c*) Voltage-clamp currents for a family of different depolarizations from −70 mV, as indicated, showing a small inward Ca_V_ current and a long-lasting K_V_ current activated by depolarization. Inset shows the inward current in more detail. (*d*) Corresponding responses of the model. (*e*) (i) Action potential firing in the model with a bias current of +4.6 pA and application of 5 µM glutamate. (ii) The corresponding calcium current (10% of total current) through NMDA receptors. (*f*) Action potential firing rate as a function of glutamate concentration, with a constant +4.6 pA bias current. (*g*) Calcium current as a function of glutamate concentration. CaV current (red, scale at right) is much greater than NMDAR calcium current. Total NMDAR calcium current (blue scale at left) rises steadily with glutamate concentration, apart from a temporary reduction at the point where spike rate rises most rapidly. The pulse NMDAR calcium current (current greater than half-amplitude, indicated by dotted line in the lower panel of (*e*)), shows a much sharper threshold behaviour, also showing an extremely high level over a narrow range of glutamate just above the firing threshold.

To gain more quantitative insight into how NMDAR current in these mPanNET tumour cells is influenced by, and influences membrane potential in physiological magnesium, we constructed a computational model incorporating the main voltage-gated conductances (Ca_V_1/Ca_V_2, K_V_2.1) which drive spike generation in normal mouse pancreatic β cells from which mPanNETs derive [14], with magnitudes determined by our voltage-clamp measurements. The model (figure 8*b*,*d*) was able to account well for the electrical responses of cells (figure 8*a*,*c*). We then added NMDARs to the model, at a density of 11 channels per cell, and computed the spike rate (figure 8*f*) and NMDAR and CaV calcium currents (figure 8*f*), as a function of ambient glutamate concentration, injecting a small depolarizing current (4.6 pA) to bias the cell near to spike threshold. This showed that the electrical activity of the cell can be strongly controlled by the extracellular glutamate concentration, with spike rate increasing from 0 to 2 Hz as glutamate is elevated from zero to about 1.5 µM. Calcium current through CaV channels (figure 8*g*) is also sharply elevated over this range. NMDAR calcium current (taken as 10% of the total NMDAR current [48]) is much smaller than the spike-generating CaV current, but increases smoothly with extracellular glutamate concentration, except for a local dip at the point at which spike rate increases most rapidly (a consequence of reduced driving force during the most depolarized phases of action potentials, and the increased block during the afterhyperpolarizations). Calcium influx through NMDARs in these cells mediates pro-invasive signalling in part by activating CaMK-II [12], which requires cooperative binding of calcium to calmodulin, and also exhibits a switch-like nonlinearity of activation via autophosphorylation. It is relevant, therefore, to evaluate calcium influx specifically during action-potential-related pulses of NMDAR current. We integrated the calcium influx during currents exceeding half the maximum amplitude reached during pulses (figure 8*e*). This ‘pulse’ NMDAR calcium current had a much sharper onset with increasing glutamate concentration, at around 1.5 µM, rising rapidly to a local maximum (figure 8*g*, dashed blue curve). Thus, blocking only a relatively small fraction of receptors or glutamate release might, in principle, be able to reduce NMDAR signalling by a much higher proportion. Overall, the model shows that it is biophysically plausible that NMDAR activation can strongly increase activity in these cells in physiological magnesium, consistent with what we found in calcium recordings.

### 3.5. Conclusions

Using sensitive electrophysiological techniques, we have been able to verify, quantify and model the physiological mechanisms of NMDAR signalling in cancer cells derived from a highly invasive GEMM of cancer developed by Li & Hanahan [12], which involve autocrine and paracrine release of glutamate—possibly through VGLUTs, activation of functional NMDARs, depolarization and calcium influx. Thus, this study provides much new biophysical insight into how cancer cells hijack the neuronal glutamate-to-NMDAR signalling circuit. In addition, we have discovered a novel source of NMDAR-activating glutamate in the tumour environment, namely the rupture of cells associated with necrosis. While mouse PanNET cells serve as a model system for the study presented here, we envision that similar glutamate-to-NMDAR signalling pathways may exist in other tumour types, since NMDAR expression is elevated in a variety of cancers. Taken together, these findings justify further work to understand the dynamics of extracellular glutamate in the complex tumour environment. Inhibition of NMDAR and VGLUTs should be explored further as therapeutic possibilities for blocking the invasive growth of cancers with high NMDAR pathway activity.

## 4. Material and methods

### 4.1. Cell culture

The mouse βTC-B6 PanNET cell line was derived from a RIP1-Tag2 mouse model of fully backcrossed C57Bl/6 strain background, and cultured by standard methods. Cells were maintained in Dulbecco's Modified Eagle's Medium (DMEM) supplemented with 10% fetal bovine serum and 50 U ml^−1^ penicillin and 50 µg ml^−1^ streptomycin, in Thermo Scientific Nunc Easyflasks (Cat. No. 156367). All culture media and supplements were from Life Technologies. Medium was exchanged every two days, and passaging was carried out at about 30–40% confluence. Cells were plated on Falcon 35 × 10 mm cell culture dishes and maintained in culture for 1–3 days prior to recording.

### 4.2. Electrophysiology

Cells were bathed in a magnesium-free Ringer solution, containing (mM): 140 NaCl, 2.5 KCl, 2 CaCl_2_, 10 glucose, 0.01 glycine, 10 HEPES/Na, pH adjusted to 7.4 with NaOH. For whole-cell recording, patch pipettes were pulled from capillary glass (Harvard Apparatus, borosilicate glass capillaries, GC150F-7.5) and filled with a solution containing 105 K gluconate, 30 KCl, 10 HEPES/KOH, 4 ATP/Mg, 0.3 GTP Na_2_, 10 creatine phosphate/Na, pH adjusted to 7.3 with NaOH. Open pipette resistance was between 5 and 10 MΩ, and the membrane potential signal was corrected for nulling of the liquid junction potential before seal formation. Whole-cell recordings were established using an Axopatch 200A patch-clamp amplifier (Axon Instruments) using capacitative feedback mode to reduce noise, low-pass filtered at 5 kHz (Bessel, 8-pole) and sampled at 20 kHz or 50 kHz with 16-bit resolution using a National Instruments X-series PCIe board and custom software written in Matlab/C++. Further low-pass Gaussian digital filtering at corner frequencies between 500–1000 Hz was applied offline. Glutamate and NMDA responses were measured by pressure ejection of agonist dissolved in the Ringer solution, through pipettes with tip diameters of 10–20 µm, and pressures of 5–10 mBar, using a custom computer-controlled pressure ejection system. Recording and perfusion pipettes were positioned with LM-Mini stepper-motor-controlled micromanipulators (Luigs and Neumann, Ratingen, Germany). Nystatin perforated patch recordings were carried out as in Akaike & Harata [49], back-filling pipettes with 150 mM CsCl, 10 mM HEPES and 150 µg ml^−1^ nystatin (pH 7.2), after dipping tips in the same solution without nystatin. All recordings were carried out at room temperature (23°C).

### 4.3. Intracellular calcium measurement

To record calcium signals, cells were loaded with the fluorescent indicator Oregon Green 488 BAPTA-1 AM (Life Technologies) at 5 µM for 1 h, and imaged using epifluorescence (Olympus IX71 microscope, UMPlan FI 10× objective, X-Cite 120 light source, EXFO Photonic Solutions), and a sCMOS camera (Zyla 4.1 CL10, Andor). Using custom programs built with the Matlab Image Processing Toolbox, cell regions were selected, and the average signals across pixels in each region were analysed as the change in fluorescence (Δ*F*) relative to the baseline level (*F*), i.e. Δ*F/F*. Cells were bathed in the same Ringer solution used for electrophysiology (see above), with or without addition of 1 mM MgCl_2_. In some experiments, a near-physiological temperature (35°C) was achieved using power resistors to heat the microscope stage, and monitored by a thermocouple in the recording bath.

### 4.4. Single channel analysis

Channel currents were measured by binning the sampled waveforms, and fitting amplitude histograms to a probability density function consisting of a sum of Gaussian distributions, by least-squares:4.1
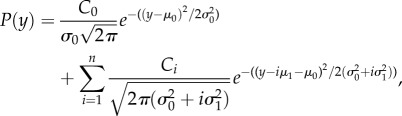
where 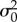 is the variance of current in the baseline, 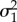 is the variance of open channel current noise for a single channel, *μ*_0_ is the baseline current level, *μ*_1_ is the single channel current amplitude, and *n* is the maximum number of channels simultaneously open. To determine the number of channels *N* and their open channel probability, the binomial distribution was used for the probability of *k* channels being simultaneously open:
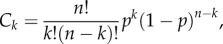
substituted into equation (4.1).

Channel gating transitions were determined by crossing of the 50% threshold amplitude between open and closed levels. Dwell-time distributions were fitted by probability density functions which were sums of exponential components:
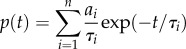
by maximum likelihood, applying a scaling correction for missed short dwell times due to the low-pass filter.

### 4.5. Capacitance measurement

Membrane capacitance was measured continuously from the phase lead of current responses to a sinusoidal membrane potential command signal in whole-cell voltage-clamp [29,50]. If 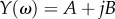 is the complex admittance of the equivalent circuit consisting of the membrane resistance *R*_m_ and *C*_m_ in parallel, in series with the access resistance *R*_a_, then
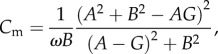
where 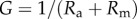. The sinusoidal voltage command signal had a frequency of 1 kHz, amplitude of 12.5 mV (peak-to-peak 25 mV) and offset of −90 mV.

### 4.6. Shikonin-induced glutamate release assay

Shikonin (Sigma, S7576) was dissolved in DMSO at a concentration of 34.8 mM, then diluted to a final concentration of 20 µM in a suspension of cells (300 000 cells ml^−1^) in Hank's Balanced Salt Solution (HBSS). After 90 min of incubation at 37°C, the cells and cell fragments were spun down and glutamate concentration was measured in the supernatant, using an Amplex Red glutamic acid assay kit (Invitrogen) and a Fluostar Omega microplate reader (BMG Labtech, Ortenberg, Germany), with excitation at 544 nm and fluorescence detection at 590 nm. A second-order polynomial function was fitted by least squares to measurements from known glutamate standard solutions, and inverted to yield glutamate measurements from the fluorescence intensity measurements.

### 4.7. Glutamate diffusion model

The diffusion equation 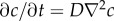, describing the concentration *c* of glutamate around a cell, was solved numerically in two dimensions, using a finite-difference representation with spatial compartments of 50 × 50 nm. The system of differential equations was solved explicitly in space and implicitly in time with an adaptive time step (Matlab ode15s), similarly to the Crank–Nicholson method. *D*, the diffusion constant for glutamate, was set at 0.5 µm^2^ ms^−1^ [51]. The surface of the cell emitted glutamate at a constant rate *q*, usually set to 10^−8^ fmoles per µm^2^ of membrane surface per ms. Concentration was clamped to zero at the upper boundary, simulating an absorbing well-stirred glutamate-free bath. The bottom (substrate) and side boundaries were reflecting. Solutions were evaluated to a time (10 s) by which an effectively steady-state distribution of glutamate was reached. The numerical solution method was validated by its exact correspondence with the analytical solution for the case of constant release of glutamate from a planar source [52]. For prediction of necrosis-induced glutamate transients (figure 6), we used the following analytical solution for release of a quantum *M* of glutamate at a single point on a planar surface, at a distance *r*:
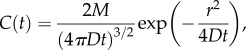
where *M* = 2 × 10^−14^ moles of glutamate at a distance *r* = 80 µm, and diffusion coefficient *D* = 0.5 µm^2^ ms^−1^. This quantity corresponds to a cell with a volume of 2 pl, containing 10 mM l-glutamate.

### 4.8. Modelling of membrane currents

A single-compartment electrical model of a cell was specified from our recorded passive electrical parameters and voltage-clamp data for active currents (figure 8), and previously-described Hodgkin–Huxley kinetic models for Ca_V_ and K_V_2.1 currents: The following set of simultaneous ordinary differential equations was used. For membrane potential *V*:
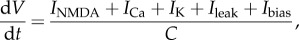
where the cell capacitance *C* was set to 16.5 pF. The NMDA receptor current was given by
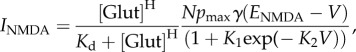
where the Hill coefficient *H* was 1.5, *K*_d_ was 1.5 µM, *p*_max_ was 0.1 (open probability when fully activated), and the total number of channels *N* in the cell was 200. *K*_1_ was 0.33 (equivalent to 1 mM Mg^2+^) and *K*_2_ was 0.06 [53]. The calcium current was as described in Migliore *et al*. [54]:
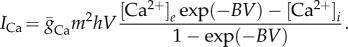


With *B* = 0.07486 mV^−1^, [Ca^2+^]*_i_* = 50 nM, [Ca^2+^]*_e_* = 1 mM, and
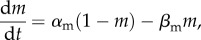
where 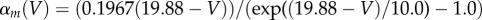, and 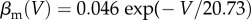 (units of ms^−1^).
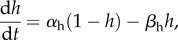
where 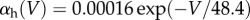 and *β*_h_(*V*) = 1/ 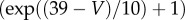.

K_V_2.1 current was modelled as described in VanDongen *et al*. [55]:
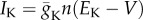

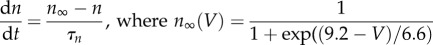

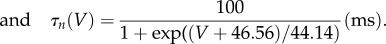
All Matlab code used in simulations is given in the electronic supplementary material.

## Supplementary Material

Supplementary Code

## Supplementary Material

Supplementary Figure 1

## Data accessibility

No data deposition is applicable for this paper. A video and all modelling code are provided as electronic supplementary material.
