# Supplementary material for: Autocrine, paracrine and necrotic NMDA receptor signalling in mouse pancreatic neuroendocrine tumour cells
Source: Open Biol. 2017 Dec 20;7(12):170221. doi: 10.1098/rsob.170221 (PMC5746548; doi:10.1098/rsob.170221)
Supplement: Supplementary Figure 1 [file rsob170221supp2.pdf]

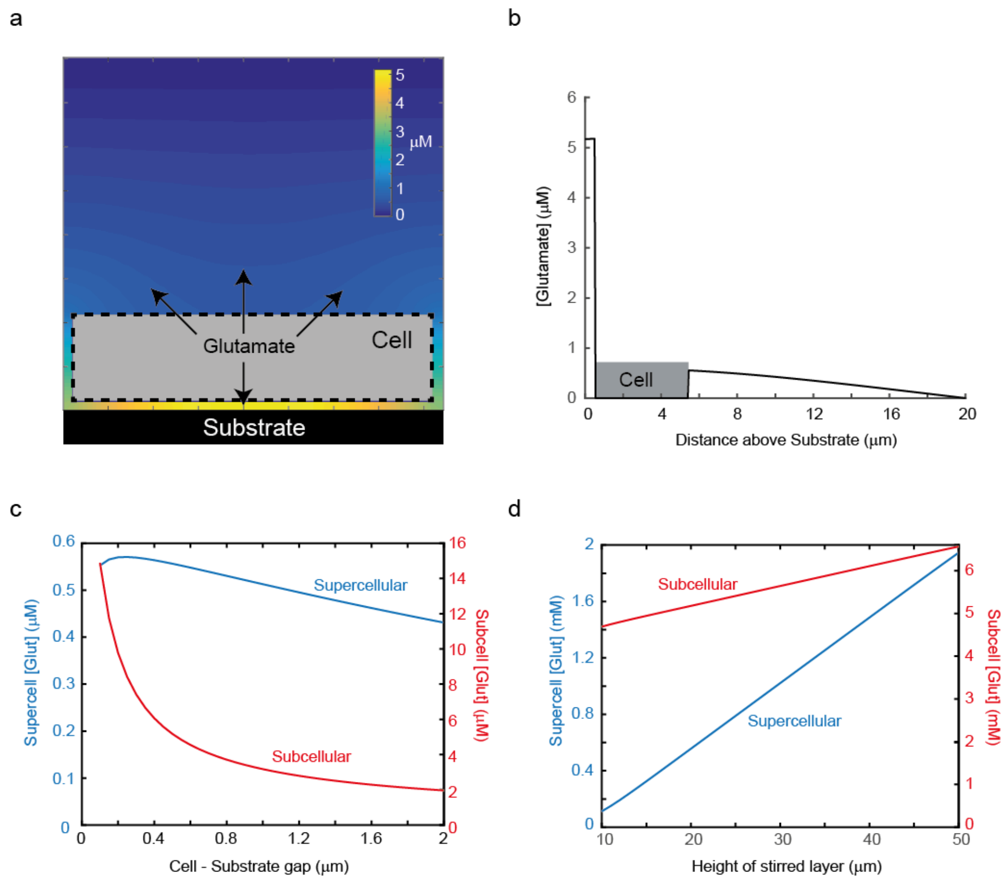

Supplementary Figure 1. (Electronic Supplementary Information). Computational model of diffusion of released glutamate. a) steady-state 2D distribution of glutamate computed for a cell which is 20 X 5 μm in cross-section, separated by a 0.5 μm gap from the underlying substrate and from reflecting side barriers representing the adjacent cells, and releases glutamate at a rate of 10^{-8} fmoles per μm^2 of membrane surface per ms. Height of stirred layer is 20 μm above substrate. b) Corresponding steady-state profile of glutamate in a section taken at the centre of the cell. c) Subcellular glutamate concentration (red curve) increases sharply as the gap between cell and substrate is reduced, while the concentration of glutamate above the cell (blue curve) is relatively insensitive to the gap distance. d) subcellular glutamate concentration is much less dependent on the height of the absorbing well-stirred layer than supercellular glutamate concentration.
